# Community-acquired diarrhea among children and adults in urban settings in Senegal: clinical, epidemiological and microbiological aspects

**DOI:** 10.1186/1471-2334-13-580

**Published:** 2013-12-09

**Authors:** Bissoume Sambe-Ba, Emmanuelle Espié, Mamadou Elimane Faye, Lassina Gadi Timbiné, Mbacké Sembene, Amy Gassama-Sow

**Affiliations:** 1 Experimental Bacteriology Unit, Pasteur Institute of Dakar, 36 avenue Pasteur, BP 220 Dakar, Senegal; 2 Epidemiology Unit, Pasteur Institute of Dakar, Dakar, Senegal; 3 University Cheikh Anta Diop, Dakar, Senegal

**Keywords:** Diarrhea, Bacteria, Virus, Parasite, Children, Adults, sub-Saharan Africa

## Abstract

**Background:**

Only limited data are available relating to the etiology of diarrhea in children and adults in Senegal. The aim of this prospective study was to describe the epidemiology and etiology of community-acquired diarrheal infections in children and adults living in urban settings.

**Methods:**

A prospective study was carried out from March 2009 to December 2010, in the urban region of Dakar, Senegal. Patients with acute diarrhea were enrolled, interviewed to collect their clinical history, and their stools were tested for bacteria, virus and parasites.

**Results:**

A total of 223 patients (including 112 children younger than five years old) with diarrhea were included. At least one enteropathogen was detected in 81% (180/223) of the patients: 29% (64/223) had bacterial infections (mainly diarrheagenic *E. coli* and *Shigella* spp), 21% (39/185) viral infections (mainly rotavirus) and 14% (31/223) parasitic infections. Co-infection was identified in 17.8% (32/180) of the patients. Viral infection was significantly more frequent in children under five years old during the dry season. Bacteria and parasites were equally frequent in all age groups. There was a seasonal variation of bacterial infections during the study period, with a higher proportion of infections being bacterial, and due to *Salmonella* spp. in particular, during the rainy season.

**Conclusion:**

Our study suggests that in urban settings in Senegal, rotavirus is the principal cause of pediatric diarrhea during the dry season and that the proportion of bacterial infections seems to be higher during the rainy season. Further work is needed to document the burden of diarrheal diseases in sub-Saharan urban communities and to identify risk factors, including those linked to the rapid and unplanned urbanization in Africa.

## Background

Diarrheal diseases remain one of the principal causes of childhood mortality and morbidity in low income countries despite significant progress in our understanding of the pathogenesis of these diseases and in their management. According to the World Health Organization, diarrheal disease is the second leading cause of death in children under five years old worldwide, and is responsible for 1.5 million child deaths every year. The risk of contracting diarrheal diseases is currently 5-fold higher in sub-Saharan Africa than in industrialized countries [1].

In sub-Saharan Africa, and particularly in Senegal, urban migration has increased over the last 30 years, resulting in a disorganized urban landscape where populations live in crowded housing conditions. This rapid expansion of cities, with the creation of urban slums, the lack of or inadequate safe water supply, inadequate drainage and sewage networks, and absence of sanitation and solid waste removal, has increased the risk of infectious diseases including diarrheal diseases and respiratory infections [2].

Most of the studies of diarrheal disease in sub-Saharan Africa over the last 20 years have focused on children under 5 years old [3-7] and rural settings [4-6,8]. Consequently, only limited information about the epidemiology and etiology of diarrheal infections in adults in developing countries is available [8-10]. Although adults are less likely to contract diarrhea, and when they do, it is unlikely to be life threatening, adults contribute to the transmission of enteric pathogens to susceptible patients, and in particular children and older people.

A wide range of bacteria, viruses, and parasites can cause diarrhea [11]. The features and the patterns of isolation of etiologic agents vary from place to place depending on the local climate and geography, and on socioeconomic factors [12]. Data on the epidemiology and etiology of diarrhea would be valuable for planning and implementing control strategies to reduce diarrhea-caused morbidity and mortality and for establishing recommendations about appropriate antimicrobial therapy in a country.

The aims of this prospective study were to describe the etiology of community-acquired diarrhea in children and adults living in a Senegalese urban setting and to assess the clinical and epidemiological characteristics of the disease.

## Methods

### Study area, target population and samples collection

**Figure 1:**
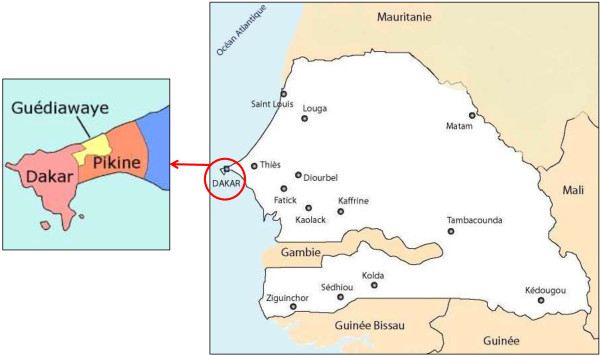
Map of Senegal and location of the three studied areas: Dakar, Pikine and Guediawaye.

The study was conducted between March 2009 and December 2010 in the urban region of Dakar, including the city of Dakar, the capital of Senegal, and two suburb cities (Pikine, Guediawaye). This region is located in the central western part of the country and covers 550 km^2^ (0.3% of the total area of the country); it has an estimated population of 3.2 million inhabitants (25% of the total population) (Figure 1). The climate is Sahelian, with a rainy season from July to October and a dry season from November to June.

Patients were recruited at the outpatient departments of eight major health centers in the three cities. The enrollment criteria were diarrhea (three or more loose or liquid stools within 24 h during the month before the visit) and no anti-infective therapy during the previous week. Patients who fulfilled these criteria were invited to participate. After providing informed consent the patient was assessed clinically by a physician and the patient, or if a child, a parent, was interviewed. The following clinical, demographic and epidemiological data were recorded: age, sex, place of residence, weight, height, symptoms such as fever (≥ 38°C), nausea, vomiting, abdominal pain, dehydration status (according to skin turgor and capillary refill), number of loose stools during the past 24 h, and whether the patient had been using antibiotic, antiparasitic, antipyretic or analgesic medication. For each patient, a stool sample was collected in sterile plastic container and immediately transported to the laboratory at the Pasteur Institute of Dakar.

### Laboratory methods

Fresh stool samples were processed and analyzed for enteric pathogens on the day of collection. For detection of bacterial pathogens (*Salmonella* spp, *Shigella* spp, *Campylobacter* spp, diarrheagenic *E. coli*, *Vibrio* spp), stools were cultured on agar plates with suitable selective media at 37°C and 42°C (for thermophilic *Campylobacter*) for periods appropriate for each microorganism. Biochemical tests and antiserum agglutination were used to identify selected isolated colonies. Unusual bacteria and *E. coli* were considered to be the sole etiologic agent of diarrhea when they were obtained as a pure culture on nonselective, solid, bromocresol-purple medium and when a significant virulence gene was identified in at least three of five colonies tested. PCR was used to detect genes coding for virulence factors (*eagg*, *eae*, *bfp*, *ipah*, *Stx1*, *Stx2*, *ST*, *LT*) as a test for diarrheagenic *E. coli*[13].

The stool specimens collected were examined microscopically for intestinal parasites (trophozoites, eggs), after staining with merthiolate-iodine-formaldehyde solution. Coccidia were detected with Kinyoun dye and Microsporidia with a modified trichrome dye with a high concentration of chromotrope 2 R.

A rapid test, based on immunochromatography, was used to detect both Rotavirus and Adenovirus (VIKIA® Rota-Adeno, bioMérieux) and PCR was used to detect Calicivirus and Enterovirus [14].

The infection was scored as a confirmed bacterial infection if one of *Salmonella* spp, *Shigella* spp, *Campylobacter* spp, diarrheagenic *E. coli* or *Vibrio* spp was isolated; as a confirmed viral infection if a rapid test for Rotavirus and Adenovirus, or a PCR for Calicivirus and Enterovirus was positive; and as a confirmed parasite infestation if *Giardia lamblia, Trichomonas intestinalis* or *Cryptosporidium* spp was detected.

### Statistical analysis

R software was used for statistical analysis [15]. The chi-square test and the Fisher exact test were used for comparison of categorical variables and the Mann–Whitney U test was used for continuous variables. A p-value < 0.05 was considered statistically significant.

Patients with confirmed infection were compared to patients without confirmed infection in terms of gender, age group (≤ 5 years, 6–15 years and > 15 years), season of diarrhea occurrence (dry and rainy season) and clinical symptoms (including hospitalization).

### Ethical aspects

The study protocol was approved by the national ethics committee for research in health (Ministry of Health of Senegal). Informed written consent was obtained from the patient, or the parent if the patient was a child, before inclusion in the study.

## Results

From March 2009 to December, 2010, 223 patients who met the enrollment criteria were included in the study: 103 (46.2%) in Pikine, 66 (29.6%) in Dakar and 54 (24.2%) in Guediawaye; 44 patients (19%) eligible for the study were excluded because they reported previous intake of antibiotic or antiparasitic drugs.

### Epidemiological and clinical characteristics

In the study population, 46.2% (103/223) were female, the median age was 5 years [IQR, 2–27] and 50.2% (112/223) of the patients were under five years old (Table 1). Almost all the patients were inhabitants of the study area (98%; 218/223), and the remaining five patients were living in the study area at the onset of diarrhea although their permanent residence was elsewhere.

**Table 1:** Epidemiological and clinical characteristics of the 223 patients with diarrhea, 2009–2010, urban setting, Senegal

	**Number, n (%)**	
	**All sample**	**Children ≤ 5 years**
	**N = 223**	**N = 112**
Age^1^*(years)*	16.4 [0.1–87]	1.7 [0.1–5]
Female	103 (46.2)	45 (40.2)
Symptoms reported by patients		
Fever	54 (25.8)	30 (26.8)
Vomiting	80 (36.5)	45 (41.3)
Dehydration	34 (16.3)	27 (26.0)
Abdominal pain	156 (72.6)	64 (60.9)
Weakness	85 (41.9)	32 (32.6)
Acute diarrhea (≤ 7 days)	184 (82.5)	86 (76.8)
Duration of diarrhea^1^*(days)*	3.7 [0–28]	4.6 [0–28]
Hospitalization	29 (13.0)	12 (10.7)
Aspect of stools		
Loose	88 (39.5)	45 (40.2)
Semi-formed	67 (30.0)	33 (29.5)
Formed	5 (2.2)	2 (1.8)
Blood	30 (13.4)	8 (7.1)
Mucus	83 (37.2)	50 (44.6)
Period		
Rainy season	143 (64.1)	65 (58)
Dry season	80 (35.9)	47 (42)
Confirmed enteric infection	72 (32.3)	46 (41.1)
Bacterial infection	64 (28.7)	27 (24.1)
Viral infection^2^	39 (21.1)	37 (33.3)
Parasitic infection	31 (13.9)	13 (11.6)

^1^Mean and range; ^2^Only 185 samples were tested for enteric viruses.

As reported by patients, the mean duration of diarrheal symptoms before visiting the health center was 3.7 days [range, 0–28 days]. The symptoms reported most frequently were abdominal pain (72.6%, 156/215), weakness (41.9%, 85/203), and vomiting (36.5%, 80/219). On examination, 25.8% (54/209) of the patients were found to be febrile and 16.3% (34/209) showed dehydration. Direct observation showed presence of mucus in 37.2% of the stools (83/223), and blood in 13.4% (30/223). Persistent diarrhea (duration > 14 days) was reported for 3.8% (8/223) of patients, including six children less than five years old. Twenty-nine of the 223 patients (13%) were hospitalized; no deaths were reported.

### Identification of enteric pathogens

No pathogen was identified or isolated from 43 (19.3%) of the 223 patients. A total of 257 potential enteric pathogens were isolated from the other 180 stool samples: 115 (44.7%) were parasites, 87 (33.8%) were bacteria and 54 (21.0%) were viruses (Table 2).

**Table 2:** Frequency and age distribution of enteric pathogens isolated from stools samples of the 223 patients with diarrhea, 2009–2010, urban setting, Senegal

**Enteric pathogens**	**No. of enteric pathogens identified (%)**		
	**All age**	**≤ 5 year**	**> 15 years**
Total (N) no. of pathogens^1^	257	141	78
Parasites	115 (44.7)	51 (36.2)	47 (60.2)
*Ascaris lumbricoides*	38 (14.8)	12	23
*Enterobius vermicularis*	26 (10.1)	21	0
*Schistosoma mansoni*	16 (6.2)	3	13
*Trichomonas intestinalis*	12 (4.7)	3	5
*Cryptosporidium* spp	11 (4.3)	5	6
*Giardia lambia*	8 (3.1)	5	0
*Microsporidium* spp	4 (1.6)	2	0
Bacteria	87 (33.8)	39 (27.6)	31 (39.7)
*Shigella* spp	28 (10.9)	8	17
Enteropathogenic *E. coli*	28 (10.9)	15	3
EAEC (*eagg*)	3	3	0
ETEC (*LT, ST*)	3	3	0
EPEC (*eae, bfp*)	21	9	3
VTEC (*stx2*)	1	0	0
*Salmonella* spp	12 (4.7)	6	4
*Citrobacter freundii*	7 (2.7)	3	4
*Klebsiella* spp	5 (2.7)	1	3
*Morganella morganii*	3 (1.9)	2	0
*Campylobacter* spp	1 (0.4)	1	0
*Aeromonas* spp	1 (0.4)	1	0
Others^2^	2 (0.8)	2	0
Viruses	54 (21.0)	51 (36.2)	0
Rotavirus	27 (10.5)	26	-
Adenovirus	17 (6.6)	17	-
Calicivirus	7 (2.7)	6	-
Enterovirus	3 (1.2)	2	-

^1^Stools samples from some patients yielded > 1 enteric pathogen.

^2^P*rovidencia rettgeri, Enterobacter cloacae.*

*Shigella* spp and pathogenic *E. coli* were the most frequently isolated bacteria (32.2%, 28/87); the unusual or opportunistic bacteria *Citrobacter freundii, Klebsiella* spp*, Morganella morganii, Aeromonas* spp*, Providencia rettgeri,* and *Enterobacter cloacae* were also found (20.7%; 18/87). Twenty-two (78.6%) of the 28 strains of *Shigella* spp were *Shigella flexneri*. Twenty-one (75%) of the 28 pathogenic *E. coli* were EPEC and EAEC, ETEC and VTEC were also found. There were 12 *Salmonella enterica* isolates including the following serotypes: Enteritidis (n = 3), Typhimurium (2), Typhi (2), Hillingdon (1), Poona (1), Putten (1), Nima (1), and Somone (1). *Ascaris lumbricoides* (38/115, 31.4%) was the most frequently identified parasite, and rotavirus (50.0%, 27/54) the most frequent enteric virus.

Thirty-two specimens yielded more than 1 potential enteric pathogen: these co-infections included parasites and bacteria (n = 8), parasites and virus (n = 3), and bacteria and viruses (n = 5), but also two different viruses (n = 14), and two different bacteria (n = 2). The pathogens most frequently involved in co-infection were pathogenic *E. coli* for bacteria, *Trichomonas intestinalis* for parasites and rotavirus for viruses. In one 22 month-old patient, co-infection with EAEC-G*iardia lambia*-Calicivirus was identified.

### Confirmed infected patients and clinical and epidemiological features

No statistically significant association was found between parasitic infection and epidemiological or clinical characteristics.

Bacterial infection occurred more frequently during the rainy season (35.7% *vs.* 16.2%, p =0.002) (Table 3). *Salmonella* infection occurred more frequently during the rainy season (7.5% *vs.* 1.2%, p =0.03). *Shigella* species were significantly more frequently isolated from patients over 15 years old (70.8% *vs.* 27.5%, p <0.001) and from cases with bloody stools (p <0.001).

**Table 3:** **Characteristics**^**1 **^**of the study participants in relation to confirmed infection with the different enteropathogens**

	**No**	**Bacteria, n (%)**	**p-value**	**Virus, n (%)**	**p-value**	**Parasite, n (%)**	**p-value**
Gender							
Male	120	39 (32.5)	0.18	23 (19.2)	0.48	19 (15.8)	0.37
Female	103	25 (24.3)		16 (15.5)		12 (11.6)	
Age range							
≤ 5 years	112	27 (24.1)	0.19	37 (33.0)	<0.01	13 (11.6)	0.28
6–15 years	30	13 (43.3)		2 (6.7)		7 (23.3)	
> 15 years	81	24 (29.6)		0		11 (13.6)	
Season							
Dry	80	13 (16.2)	0.002	21 (26.2)	0.01	10 (12.5)	0.65
Raining	143	51 (35.7)		18 (12.6)		21 (14.7)	

^1^No statistically significant association was found between any confirmed infection and clinical characteristics.

Viral infection was significantly associated with age: a greater proportion of children younger than five years old than older patients were infected with virus (33% *vs.* 1.8%, p <0.001). A significantly greater proportion of infections during the dry season than rainy season were viral (31.8% *vs.* 15.2%, p <0.01).

## Discussion

To our knowledge, this study is the first study in a sub-Saharan country, including Senegal, investigating the etiology and clinical and epidemiological characteristics of diarrhea in patients, both children and adults, living in urban settings.

At least one enteric pathogen was isolated from stools from 81% of patients with diarrhea. The enteric pathogens isolated from these cases of diarrhea were most frequently bacteria (29%), although both virus (21%) and parasites (14%) were also isolated from many cases. The corresponding values for children younger than five years old were similar except that viral infection was more frequent. These values are higher than those reported by studies conducted in rural African areas [3-5] and urban African settings [16].

The etiological agent was most frequently a bacterium in our study, with diarrheagenic *E. coli* and *Shigella* spp being predominant. These findings are consistent with published results [5,7,8,16]. However, in contrast with previous studies [3,5], the proportion of bacterial infections seemed to be higher during the rainy season. In the Dakar region, the rainy season includes the warmest months and the period of floods. Flooding is responsible for poor sanitation and increases human contact with wastewater; it is also associated with cholera outbreaks [17]. Therefore, it is likely that a large number of diarrheal episodes during the rainy season results from increased exposure to environmental pathogens and contaminated food. Only one isolate of VTEC and another of *Campylobacter* were isolated from children less than 15 years old during this study. This suggests that the occurrence of VTEC and *Campylobacter* infections in Senegal is similar to those described in Ghana and Tanzania [5,6], and lower than those observed in some other African countries (Kenya, Nigeria and Mozambique) [4,7,18,19]. Eighteen unusual or opportunistic bacteria were also isolated from patients with diarrhea. These findings are consistent with reports from studies in Burkina Faso [20] and in Senegal, of HIV-infected patients [9]. Unfortunately, no data was recorded about HIV status, co-morbidities or concomitant disease (*e.g.* malaria, acute respiratory infection or malnutrition).

Our study also showed that rotavirus and other virus seem to be significant pathogens among children younger than five years, especially among children younger than one year, confirming their role already described in the occurrence of pediatric diarrhea [21]. Furthermore, a higher frequency of viral infection was observed during the dry and cold period, thereby demonstrating what it’s already observed in other sub-Saharan countries [3,5,6].

Parasites were detected in 14% of all patients and in 12% in children younger than five years. This proportion is higher in comparison to those described in studies conducted in other developing countries [4,6,22]. *Schistosoma mansoni* infection was detected mainly in adults. For these patients who reported a previous stay in the North of Senegal, the origin of the contamination was probably linked to their occupation (i.e. farmers) in the Senegal River Basin, which is known to be an endemic area for bilharzias [23], but no further investigation was carried out to confirm this hypothesis.

In this study, one third of the patients with diarrhea were found to have co-infections, involving more than one potential enteropathogen. In developing countries, it is not uncommon to isolate more than one enteric pathogen from the same patient. The prevalence of co-infection in our patients younger than five years old was higher than reported in Burkina Faso (10.0%) [3] and Madagascar (22.8%) [24]. The relative contributions of each individual enteropathogen to the clinical symptoms cannot be determined in such cases of co-infection. Indeed, the interpretation of data on co-infections is complicated because not all the potential enteropathogens detected necessarily contribute to the etiology of the patient’s diarrheal disease. Due to the limited sample size and the study design, we were not able to analyze indicators of severe diarrhea in patients with co-infections (*e.g*., admission to an intensive care unit, or death) that would have prompted more extensive investigation.

Our study has several limitations, including the study design: the population included was not a random sample and of limited size. Stool samples from a comparable control population were not studied so the extent of asymptomatic carriage of enteropathogens cannot be evaluated. The absence of controls in this study prevents testing for associations between particular organisms and the occurrence of diarrhea. Our study describes the prevalence of diarrheal diseases among patients seeking care, and this may not represent the burden of disease in the general urban population. The population studied included three times as many children less than five years old than the general population [25], probably because the threshold for seeking care is lower for young children with diarrhea than for older patients. The small sample size was due, in part, to difficulties recruiting patients who met the inclusion criteria: about one fifth of patients were excluded because they reported having recently taken antibiotics or antiparasitic drugs.

## Conclusion

This study suggests that in urban settings in Senegal, diarrheal infections are mainly associated with enteric bacteria display a marked seasonality, being most frequent during the rainy season. In children younger than five years old, viral infections, with rotavirus in particular, are predominant, and are more frequent during the dry season. The urban context with its particular living conditions (including uncontrolled expansion of urban slums and informal settlements, residential overcrowding, or environmental degradation) may explain these patterns.

Further studies, including more representative samples or with control group or both, are needed to determine the burden of diarrheal diseases in sub-Saharan urban communities. Such studies may identify specific risk factors linked to the rapid and unplanned urbanization in Africa.

## Competing interests

The authors declare that they have no competing interest.

## Authors’ contribution

AGS designed the study. BSB was responsible for recruitment, interview of the patients, sample collection and transport to the laboratory. BSB, FME and TLG were in charge of laboratory procedures. EE did the statistical analysis. BSB, EE and AGS wrote the manuscript. All authors read and approved the final manuscript.

## Pre-publication history

The pre-publication history for this paper can be accessed here:

http://www.biomedcentral.com/1471-2334/13/580/prepub
